# Supplementary material for: nnSVG for the scalable identification of spatially variable genes using nearest-neighbor Gaussian processes
Source: Nat Commun. 2023 Jul 10;14:4059. doi: 10.1038/s41467-023-39748-z (PMC10333391; doi:10.1038/s41467-023-39748-z)
Supplement: Supplementary file 3 — Reporting Summary [file 41467_2023_39748_MOESM3_ESM.pdf]

nature portfolio

Corresponding author(s): Dr. Stephanie Hicks

Last updated by author(s): Jun 14, 2023

Reporting Summary

Nature Portfolio wishes to improve the reproducibility of the work that we publish. This form provides structure for consistency and transparency in reporting. For further information on Nature Portfolio policies, see our Editorial Policies and the Editorial Policy Checklist.

Statistics

For all statistical analyses, confirm that the following items are present in the figure legend, table legend, main text, or Methods section.

n/a	Confirmed	
☐	☒	The exact sample size (*n*) for each experimental group/condition, given as a discrete number and unit of measurement
☐	☒	A statement on whether measurements were taken from distinct samples or whether the same sample was measured repeatedly
☐	☒	The statistical test(s) used AND whether they are one- or two-sided<br>*Only common tests should be described solely by name; describe more complex techniques in the Methods section.*
☐	☒	A description of all covariates tested
☐	☒	A description of any assumptions or corrections, such as tests of normality and adjustment for multiple comparisons
☐	☒	A full description of the statistical parameters including central tendency (e.g. means) or other basic estimates (e.g. regression coefficient) AND variation (e.g. standard deviation) or associated estimates of uncertainty (e.g. confidence intervals)
☐	☒	For null hypothesis testing, the test statistic (e.g. *F*, *t*, *r*) with confidence intervals, effect sizes, degrees of freedom and *P* value noted<br>*Give P values as exact values whenever suitable.*
☒	☐	For Bayesian analysis, information on the choice of priors and Markov chain Monte Carlo settings
☒	☐	For hierarchical and complex designs, identification of the appropriate level for tests and full reporting of outcomes
☐	☒	Estimates of effect sizes (e.g. Cohen's *d*, Pearson's *r*), indicating how they were calculated

Our web collection on statistics for biologists contains articles on many of the points above.

Software and code

Policy information about availability of computer code

Data collection	nnSVG is freely available as an R package from Bioconductor (as of 2023-06-14: nnSVG version 1.4.1 available in Bioconductor release version 3.17) at https://bioconductor.org/packages/nnSVG. The package is also available from GitHub at https://github.com/lmweber/nnSVG. Code to reproduce all preprocessing, analyses, and figures in this manuscript is available from GitHub at https://github.com/lmweber/nnSVG-analyses. We used nnSVG version 1.3.10 for the analyses in this manuscript.
Data analysis	nnSVG is freely available as an R package from Bioconductor (as of 2023-06-14: nnSVG version 1.4.1 available in Bioconductor release version 3.17) at https://bioconductor.org/packages/nnSVG. The package is also available from GitHub at https://github.com/lmweber/nnSVG. Code to reproduce all preprocessing, analyses, and figures in this manuscript is available from GitHub at https://github.com/lmweber/nnSVG-analyses. We used nnSVG version 1.3.10 for the analyses in this manuscript.

For manuscripts utilizing custom algorithms or software that are central to the research but not yet described in published literature, software must be made available to editors and reviewers. We strongly encourage code deposition in a community repository (e.g. GitHub). See the Nature Portfolio guidelines for submitting code & software for further information.

Data

Policy information about availability of data

All manuscripts must include a data availability statement. This statement should provide the following information, where applicable:

- Accession codes, unique identifiers, or web links for publicly available datasets
- A description of any restrictions on data availability
- For clinical datasets or third party data, please ensure that the statement adheres to our policy

The datasets used for the analyses in this manuscript can be downloaded in SpatialExperiment format (Righelli et al. 2022) from the STexampleData Bioconductor package (Weber et al. 2022), which includes annotation labels from the original sources, and the spatialLIBD Bioconductor package (Pardo et al. 2022). The original datasets and annotations are sourced from Maynard and Collado-Torres et al. (2022) and Pardo et al. (2022) (Visium human DLPFC dataset), Stickels et al. (2020) and Cable et al. (2021) (Slide-seqV2 mouse HPC dataset), Stahl et al. (2016) (ST mouse OB dataset), and Lohoff and Ghazanfar et al. (2021) (seqFISH mouse embryo dataset). Source data are provided with this paper.

Human research participants

Policy information about studies involving human research participants and Sex and Gender in Research.

Reporting on sex and gender	Previously published publicly available datasets were used for all analyses in this manuscript.
Population characteristics	n/a
Recruitment	n/a
Ethics oversight	n/a

Note that full information on the approval of the study protocol must also be provided in the manuscript.

Field-specific reporting

Please select the one below that is the best fit for your research. If you are not sure, read the appropriate sections before making your selection.

- [x] Life sciences
- [ ] Behavioural & social sciences
- [ ] Ecological, evolutionary & environmental sciences

For a reference copy of the document with all sections, see nature.com/documents/nr-reporting-summary-flat.pdf

Life sciences study design

All studies must disclose on these points even when the disclosure is negative.

Sample size	Previously published publicly available datasets were used for all analyses in this manuscript. The number and selection of datasets was chosen to cover a representative sample of widely used spatially-resolved transcriptomics platforms to ensure that method performance was robustly demonstrated across platforms.
Data exclusions	No data were excluded from the analyses.
Replication	Replication was not used since the methods described in this study are intended for exploratory and unsupervised analyses, and no original experimental data were generated for this study.
Randomization	Randomization was not used since the methods described in this study are intended for exploratory and unsupervised analyses, and no original experimental data were generated for this study.
Blinding	Blinding was not used since the methods described in this study are intended for exploratory and unsupervised analyses, and no original experimental data were generated for this study.

Reporting for specific materials, systems and methods

We require information from authors about some types of materials, experimental systems and methods used in many studies. Here, indicate whether each material, system or method listed is relevant to your study. If you are not sure if a list item applies to your research, read the appropriate section before selecting a response.

Materials & experimental systems

n/a	Involved in the study
☒	☐ Antibodies
☒	☐ Eukaryotic cell lines
☒	☐ Palaeontology and archaeology
☒	☐ Animals and other organisms
☒	☐ Clinical data
☒	☐ Dual use research of concern

Methods

n/a	Involved in the study
☒	☐ ChIP-seq
☒	☐ Flow cytometry
☒	☐ MRI-based neuroimaging
